# Is the Alpha Variant of SARS-CoV-2 Associated with a Higher Viral Load than the Historical Strain in Saliva Samples in Patients with Mild to Moderate Symptoms?

**DOI:** 10.3390/life12020163

**Published:** 2022-01-21

**Authors:** Camille Bonnet, Shirley Masse, Hayat Benamar, Ana-Maria Vilcu, Morgane Swital, Thomas Hanslik, Sylvie van der Werf, Xavier Duval, Fabrice Carrat, Alessandra Falchi, Thierry Blanchon

**Affiliations:** 1 Sorbonne Université, INSERM, Institut Pierre Louis d’Epidémiologie et de Santé Publique (IPLESP), 75012 Paris, France; hayat.benamar@iplesp.upmc.fr (H.B.); ana-maria.vilcu@iplesp.upmc.fr (A.-M.V.); morgane.swital@iplesp.upmc.fr (M.S.); thomas.hanslik@iplesp.upmc.fr (T.H.); fabrice.carrat@iplesp.upmc.fr (F.C.); thierry.blanchon@iplesp.upmc.fr (T.B.); 2 UR7310 Bioscope, Université de Corse Pascal Paoli, 20250 Corte, France; masse_s@univ-corse.fr (S.M.); falchi_a@univ-corse.fr (A.F.); 3 UFR de Médecine, Université de Versailles Saint-Quentin-en-Yvelines, 78000 Versailles, France; 4 Assistance Publique—Hôpitaux de Paris (APHP), Hôpital Ambroise Paré, Service de Médecine Interne, 92100 Boulogne Billancourt, France; 5 Institut Pasteur, Université de Paris, Molecular Genetics of RNA viruses Unit, CNRS UMR 3569, F-75015 Paris, France; sylvie.van-der-werf@pasteur.fr; 6 Institut Pasteur, Université de Paris, National Reference Center for Respiratory Viruses, F-75015 Paris, France; 7 Centre d’Investigation Clinique, AP-HP, Hôpital Bichat, INSERM CIC 1425, F-75018 Paris, France; xavier.duval@bch.aphp.fr; 8 IAME, INSERM, Université de Paris, F-75018 Paris, France; 9 Unité de Santé Publique, AP-HP, Hôpital Saint-Antoine, 75012 Paris, France

**Keywords:** COVID-19, SARS-CoV-2, variant, primary care, viral load

## Abstract

During the COVID-19 pandemic, several generic variants emerged, including the Alpha variant, with increased transmissibility compared to historical strains. We aimed to compare the evolution of the viral load between patients infected with the Alpha variant and those infected with the historical SARS-CoV-2 strains, while taking into account the time interval between the onset of symptoms and samples. We used data collected from patients with an acute respiratory infection (mild to moderate symptoms) and seen in consultation in primary care, included in a prospective longitudinal study, COVID-A. Patients performed four salivary samples during the follow-up. All patients who had at least one of the saliva samples test positive for SARS-CoV-2 were included in the analysis. Overall, 118 patients were included: 89 infected by the historical strain and 29 infected by the Alpha variant. Even though we tended to observe a higher viral load in the Alpha variant group, we found no significant difference in the evolution of the viral load in saliva samples between patients infected with the Alpha variant of the SARS-CoV-2 and those infected by historical strains when controlling for the time interval between the onset of symptoms and sampling.

## 1. Introduction

In the midst of the COVID-19 pandemic, various genetic variants of SARS-CoV-2 gradually emerged. Among them, the Alpha variant (B.1.1.7 or 20I/501Y.V1), initially detected in the United Kingdom at the end of 2020, spread rapidly in France to become the dominant circulating strain from the months of March to June 2021 [1]. The Alpha variant possesses a large number of non-synonymous substitutions of immunological importance [2]. It also presents a deletion at positions 69 and 70 of the spike protein (Δ69–70) that is linked to immune escape in immunocompromised patients [3] and has been associated with the failure of diagnostic tests using the ThermoFisher TaqPath probe, which targets the spike protein [4].

The emergence of new variants of concern (VOCs), such as the Alpha variant, poses challenges in terms of epidemic control [5]. Many studies reported that the Alpha variant was associated with a higher frequency of severe forms and mortality than historical strains [6]. Using almost 2,000,000 SARS-CoV-2 sequences from the Global Initiative On Sharing All Influenza Data (GISAID) database, Campbell et al. estimated that the Alpha variant had 29% increased transmissibility compared to the historical strain [7]. In a study using data from over 80,000 community-based SARS-CoV-2 tests in England between 1 October 2020 and 16 January 2021, it was also shown that the Alpha variant had increased transmissibility compared to other lineages, with an estimated 50–100% higher reproduction number [8].

Considering the association between viral load and transmissibility [9], information on specific virologic parameters such as a viral load or duration of viral shedding according to the SARS-CoV-2 variant is crucial to better understand the differences in infectiousness and, thus, to adapt the barrier measures to limit their spread [10]. Using data from over 25,000 subjects in Germany (including inpatients and pre-symptomatic, asymptomatic, or mild-symptomatic patients from walk-in testing centers), Jones et al. found that individuals infected with the Alpha variant had higher viral loads than those infected with another strain [11]. However, these studies were carried out using laboratory screening data with little information on patients’ characteristics and did not account for the time interval between symptom onset and testing, which may impact observed viral loads. Studies on the duration of viral shedding according to SARS-CoV-2 variants in symptomatic patients consulting in primary care are necessary to complement these results.

To better understand variant-specific viral characteristics and evolution, we conducted a longitudinal prospective study among mild to moderate symptomatic SARS-CoV-2 patients consulting in primary care (general practice or pediatricians). Sampling for this study spanned the period between July 2020 and May 2021. During this time, the historical strains were initially prevalent but were replaced by the Alpha variant at the end of 2020. It then became dominant between March and June 2021. The aim of our study was to compare the evolution of the cycle threshold (Ct) value, as a proxy of the viral load, between patients infected with the Alpha variant and those infected with the SARS-CoV-2 historical strains, while accounting for the time interval between symptom onset and testing.

## 2. Materials and Methods

### 2.1. Patient Recruitment and Clinical Data Collection

**Figure 1:**
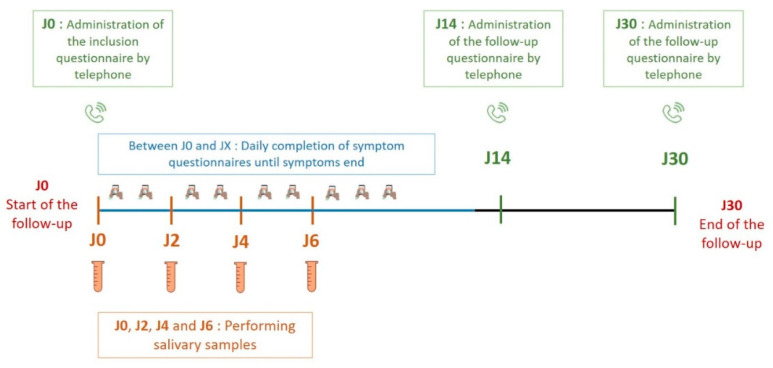
Course of patient follow-up in the COVID-A study France, 2020–2021.

We used data collected in the longitudinal prospective study, COVID-A, which aimed to provide information on clinical and virological courses of COVID-19 in patients consulting in primary care in France from 5 June 2020 to 30 June 2021. Eligible patients were those aged eight years or older, consulting a general practitioner or pediatrician for an acute respiratory infection (ARI), defined by an abrupt onset of fever (or feeling of fever) and respiratory signs, and who agreed to participate in the study. A clinical and virological follow-up of these patients was conducted. Within the virological follow-up, patients were first asked to perform a saliva self-collection in the presence of the physician during the inclusion consultation. They were then asked to take the same type of collection at home two, four, and six days after the inclusion. Thus, for each patient, we collected up to four saliva samples. An explanatory card was given to each patient to explain the procedure for collecting saliva. Clinical data were collected at inclusion by the physician, including age, sex, date of onset of symptoms, and medical history. The patient was also contacted following the consultation by telephone by a person in charge of the study to collect additional information on his socio-demographic situation and his medical care. The clinical follow-up of the patient was carried out by collecting the daily symptoms presented via a short questionnaire sent by SMS or email. The course of patient follow-up in the COVID-A study is shown in Figure 1.

### 2.2. Study Population

Our study population included all patients recruited in the COVID-A study who had at least one salivary sample that tested positive for SARS-CoV-2.

### 2.3. Virological Analysis

All saliva samples were analyzed in the same virology laboratory at the University of Corte, France. Total nucleic acid was extracted from 200 μL of saliva samples using the QIACUBE processing system with the QIAamp 96 Virus QIAcube HT kit (Qiagen, Hilden, Germany) and eluted into 100 μL of total nucleic acid. RT-qPCR was performed for each sample initially with the TaqPath COVID-19 kit (Thermo Fisher Scientific, Waltham, MA, USA). The TaqPath COVID-19 kit is a multiplex RT-qPCR diagnostic test targeting three regions of the SARS-CoV-2 genome: the open reading frame 1ab (ORF1ab), the gene for the S protein, and the gene for the N protein. Forty amplification cycles were performed by the assay. At least two genes have to be detected for the result to be reported as positive for SARS-CoV-2 with a Ct < 37. The TaqPath COVID-19 test amplifies three target genes of the SARS-CoV-2: the ORF1ab gene, N gene, and S gene. As the Alpha variant of SARS-CoV-2 has a deletion at position 69–70 of the spike protein, this results in a loss of amplification for the S gene. The absence of detection of the S gene target in an otherwise positive PCR test thus appears to be a highly specific biomarker for the B.1.1.7 lineage (the Alpha variant) [12]. An inconclusive result is reported when only one gene is detected after consecutive repeat testing. In a second step, the positive samples were analyzed by RT-qPCR using the ID SOLUTION (GRABELS, France) ID™ SARS-CoV-2/N501Y/E484K Quadruplex kit, which allows the detection of the presence of the SARS-CoV-2 virus in a single reaction and specifically targets N501Y and E484K mutations. All positive results had amplifiable MS-2 internal control, with no evidence of general inhibition in the RT-PCR reaction. Lineage information was confirmed by sequencing by a team from the Pasteur Institute in Paris. Two comparative groups were therefore formed: the Alpha variant group and the historical strain group. The ‘historical strain’ group included SARS-CoV-2 variants belonging to the following lineages (or clades): 20A Basal pandemic strain (S: 614G)—international distribution/20B/20D or 20E (EU1). Patients for whom a variant other than the Alpha and the historical were identified were excluded (*n* = 5 were infected with the Beta variant (B.1.351)). Viral load was compared by proxy using RT-PCR Ct values, estimated with the TaqPath kit.

### 2.4. Statistical Analysis

First, the characteristics of the patients included in our analysis sample were described (numbers, proportion) and compared between the two groups studied using a Chi-2 or Fisher exact test, according to the theoretical numbers in each category. Missing dates of symptoms’ onsets were imputed by randomly assigning values drawn from the distribution of observed delays between symptoms’ onset and inclusion, conditional on the variant.

Kaplan–Meier survival curves were produced to estimate the duration of positivity, defined as the time window between the patient’s onset of symptoms and the date of their first negative sample observed during the follow-up. A patient was interval-censored between the last positive and the first negative sample collected using mid-point imputation or right-censored if still positive at the last sample observed.

Then, all the saliva samples were described in terms of viral load according to the time in days between the date of onset of symptoms and the date of sampling, using the following categories: negative, Ct > 23, Ct < 23 [13]. The distribution of these categories was compared between the two groups (historical strain and Alpha variant). Finally, we conducted an analysis restricted to SARS-CoV-2 positive samples. We compared the Ct value distribution of either ORF1ab or N-target genes between the Alpha variant sample and the historical strain sample according to the time from symptom onset to the date of sample collection.

## 3. Results

We included 118 patients who had at least one of the saliva samples test positive for SARS-CoV-2: 89 infected by the historical strain and 29 infected by the Alpha variant. Overall, 424 saliva samples were analyzed: 327 in the ‘historical strain’ group and 97 in the ‘Alpha variant’ group.

Patients were included between 17 July 2020 and 20 May 2021 and their characteristics are presented in Table 1. The median age was 47 years [IQR: 30; 55]. More than half (*n* = 63, 54.3%) of the patients were women and 14.3% (*n* = 16) were smokers. About 25% (*n* = 28) of patients presented at least one comorbidity and 17.9% (*n* = 20) had a BMI of 30 or more. No differences in patient characteristics were observed between the Alpha variant and the historical strain groups.

The most frequently reported symptoms presented at least once during clinical follow-up were stuffy or runny nose (*n* = 78; 69.6%), fatigue (*n* = 76; 67.9%), cough (*n* = 72; 64.3%), and headache (*n* = 72; 64.3%). These symptoms did not differ between the two groups. Three patients were hospitalized for dyspnea: two infected by the historical strain and one by the Alpha variant, and the duration of hospitalization was between four and nine days. The median time from symptom onset to study inclusion was 2 days [q1 = 2.0; q3 = 4.0] and the median time from symptom onset to the last saliva sample was 8 days [q1 = 7.0; q3 = 10.0]. There was no significant difference between the two groups for these times (*p*-value = 0.36 and *p*-value = 0.56).

**Figure 2:**
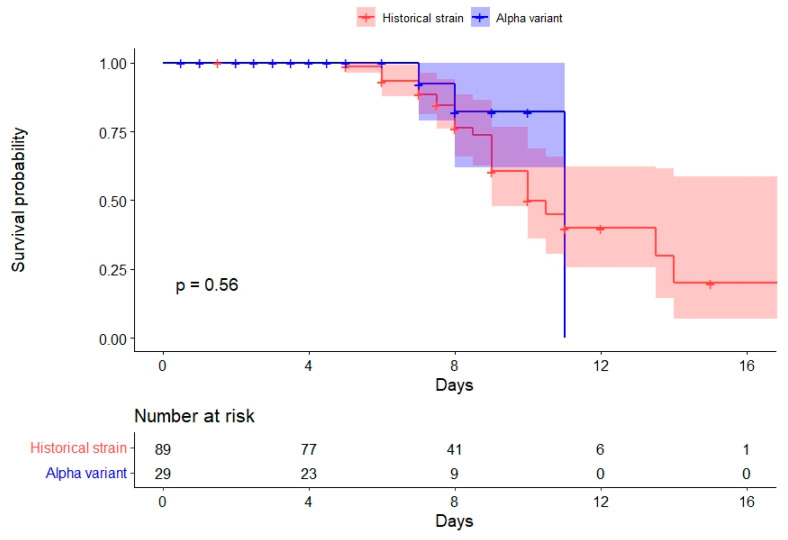
Kaplan–Meier survival curves presenting the probability of remaining SARS-CoV-2 positive, according to days since onset of symptoms and SARS-CoV-2 strain. COVID-A, France, 2020–2021.

The survival curves for duration of positivity were not significantly different between the two groups (*p*-value of log-rank test = 0.56) (Figure 2).

**Figure 3:**
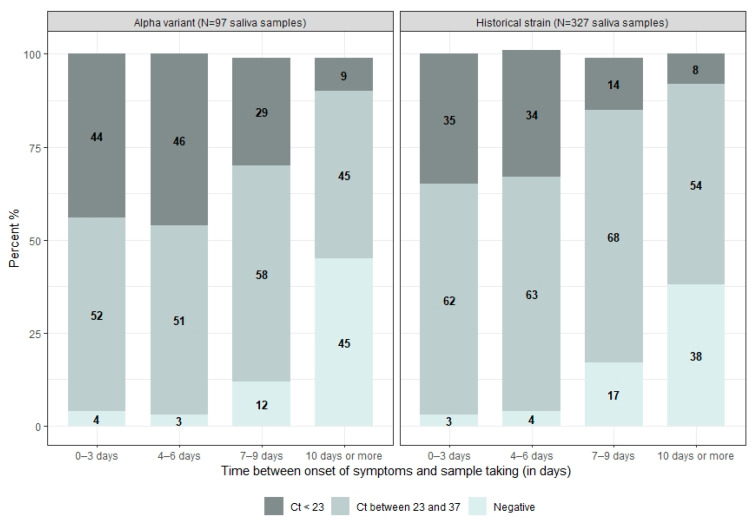
Distribution of the SARS-CoV-2 status (negative, Ct between 23 and 37, Ct < 23) by the delay (in days) between the date of onset of symptoms and the date of sampling, according to the SARS-CoV-2 strain, COVID-A, France, 2020–2021.

Regarding the distribution of the viral load in all saliva samples collected (Figure 3), in the Alpha variant group, approximately 44% (*n* = 12) of samples collected within 3 days of symptom onset had a viral load of less than 23, compared with 35% (*n* = 25) in the historical strain group (*p*-value = 0.52). Between 4 and 6 days, the proportion of samples with a viral load of less than 23 was 46% (*n* = 16) and 34% (*n* = 36) in the Alpha and historical strain groups, respectively (*p*-value = 0.39). This proportion decreased to 29% (*n* = 7) in the Alpha group and 14% (*n* = 14) in the historical strain group for samples taken between 7 and 9 days after the onset of symptoms (*p*-value = 0.25). At 10 days or more, 45% (*n* = 5) were negative in patients infected with the Alpha variant, compared with 38% (*n* = 19) in patients infected with the historical strain (*p*-value = 0.88).

**Figure 4:**
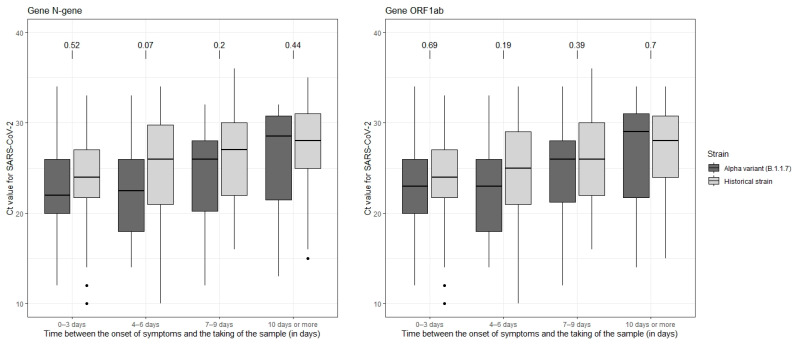
Box plot of the population of the Alpha variant group and the historical strain group within ORF1ab and N-gene according to the delay (in days) between the date of onset of symptoms and the date of sampling, COVID-A, France, 2020–2021. Median Ct is shown by a black horizontal bar. Above both plots are the results of the Student’s *t*-test. Abbreviations: Ct: cycle threshold; N: nucleocapsid; ORF1ab: open reading frame 1ab.

The evolution of Ct values for the ORF1ab and N genes observed in SARS-CoV-2 positive samples according to the time between the onset of symptoms and the sampling is presented in Figure 4. Overall, there was an increase in Ct values with increasing time since symptom onset, but the distribution of Ct values was not significantly different between the two groups in any class of time from symptom onset to sampling. These results were similar regardless of the gene.

## 4. Discussion

In a patient population with ARI symptoms who tested positive for the SARS-CoV-2 and were seen in primary care, even though we tended to observe a higher viral load in the Alpha variant group, we found no significant difference in the evolution of the Ct values (used as a proxy of viral load) in saliva samples between patients infected with the Alpha variant of the SARS-CoV-2 and those infected by historical strains when controlling for the time between the onset of symptoms and sampling. To our knowledge, this study is the first to be conducted in a population of symptomatic patients seen in primary care consultation.

Previous studies have suggested an association between the variant Alpha and a higher viral load compared to historical strains, but most studies have not addressed the time since the onset of symptoms and could not compare viral load evolution [14]. Based on surveillance data collected in an Italian region, Calistri et al. showed that Ct values for the N-gene were significantly lower in patients infected by the Alpha variant compared to those infected by other lineages [15]. Kidd et al. studied the Ct values from the nasopharyngeal samples (NPS) of 641 patients who tested positive for SARS-CoV-2 in November as part of the ‘Test and Trace’ program in the UK [16] and they concluded that samples infected with the Alpha variant are more likely to have high viral loads, but they stressed the need for further studies to confirm their findings. Teyssou et al. compared virological data obtained by NPS from 332 patients infected with historical SARS-CoV-2 strains and 249 patients infected with the Alpha variant. They showed that the viral load was 10 times higher in patients infected by the Alpha variant than in patients infected by the historical strain [17]. However, to our best knowledge, only one study took into account the notion of temporality by comparing viral loads at the time of symptom onset and a longer duration of shedding [18]. They showed a higher viral load at symptom onset in the Alpha and Beta variant patients than in historical strain patients. Although the majority of studies report consistent results, several elements limit comparisons: the study population, the clinical presentation, the reasons for sampling (diagnosis, screening, etc.), the sampling techniques, the quality of the samples, and the assay used to estimate the value of Ct. Second, in contrast to our study, the majority of studies were conducted using virological data from NPS. While salivary samples have been shown to be a good alternative for the detection of SARS-CoV-2, few data are available on the measurement of viral load in saliva and the results seem to be contradictory. In a study investigating the performance of saliva samples for the detection of the SARS-CoV-2 virus [19,20], the authors found lower Ct values in saliva specimens than in NPS [21], but other studies showed the opposite [22].

The main strengths of our study are the availability of several virological data per patient and the ability to consider the time since the onset of symptoms, thus allowing the analysis of an evolution over time. The fact that the study population was patients with mild to moderate forms of COVID-19 seen in primary care and responding to an ARI clinical definition is also a strength of this study, because most studies have been either hospital-based or based on screening data without symptom information. Finally, all samples were analyzed in the same virology laboratory, which limits biases related to the technique and interpretation of results between the two groups studied. The main limitation of our study is the small number of patients included in the Alpha variant group and the lack of statistical power. There may be a lack of homogeneity between the first sample, taken with the help of the doctor during the consultation, and those performed at home, where patients were asked to take them in the morning, before brushing their teeth or having breakfast. This can result in low comparability between Ct values from the first one and the three others. Finally, due to the study protocol and participation procedures, some age groups were underrepresented in our sample, such as young children or the elderly. It would also have been interesting to look specifically at certain age groups. A study in children in Germany showed no significant difference in viral load between patients infected with the Alpha variant and those infected with other lineages [23]. In addition, the relationship between symptom intensity and viral load is still unclear but our numbers did not allow us to conduct these analyses [24].

## 5. Conclusions

We found no significant difference in the evolution of the viral load between patients infected with the Alpha variant and those infected with the historical strain, in patients with mild to moderate COVID-19 seen in primary care consultations in France, when taking into account the time interval between symptoms’ onset and sampling. Regarding the trend of our results suggesting a higher viral load in the Alpha group, it is possible that this lack of difference is related to a lack of statistical power in our study.

## Figures and Tables

**Table 1:** Patient characteristics of study sample, COVID-A, France, 2020–2021.

	Total N = 118 *n* (%)	Historical Strain N = 89 *n* (%)	Alpha Variant N = 29 *n* (%)	*p*-Value
Inclusion period	17 July 2020–20 May 2021	17 July 2020–26 April 2021	5 February 2021–20 May 2021	
Age, in years (median [Q1; Q3]) (m.d = 2)	47 [30; 55]	46 [28; 54]	49 [38; 54]	0.36
Age group, in years (m.d = 2)				0.28
<15	4 (3.4)	3 (3.4)	1 (3.7)	
15–34	29 (25.0)	25 (28.1)	4 (14.8)	
35–54	54 (46.6)	39 (43.8)	15 (55.6)	
55 or more	29 (25.0)	22 (24.7)	7 (25.9)	
Sex (m.d = 2)				0.71
Woman	63 (54.3)	47 (52.8)	16 (59.3)	
Man	53 (44.9)	42 (47.2)	11 (40.7)	
Smoking status: smoker (m.d = 6)	16 (14.3)	13 (14.9)	3 (12.5)	0.75
Professional situation (m.d = 6)				0.42
Employed	85 (75.9)	64 (73.6)	21 (84.0)	
Other	27 (24.1)	23 (26.4)	4 (16.0)	
Obesity (BMI ≥ 30) (m.d = 6)	20 (17.9)	16 (18.4)	4 (16.0)	0.56
At least one comorbidity (m.d = 2)	28 (24.1)	24 (27.0)	4 (14.8)	0.30
Symptoms presented at least once during the clinical follow-up (m.d = 6)				
Stuffy or runny nose	78 (69.6)	60 (69.8)	18 (69.2)	0.99
Fatigue	76 (67.9)	61 (70.9)	15 (57.7)	0.30
Cough	72 (64.3)	56 (65.1)	16 (61.5)	0.92
Headache	72 (64.3)	54 (62.8)	18 (69.2)	0.71
Diarrhea or nausea/vomiting	68 (60.1)	51 (59.3)	17 (65.4)	0.74
Myalgia	61 (54.5)	46 (53.5)	15 (57.7)	0.88
Anosmia or agueusia	58 (51.8)	45 (52.3)	13 (50.0)	0.99
Fever or a feeling of fever	55 (49.1)	44 (51.2)	11 (42.3)	0.57
Lack of appetite	44 (39.3)	34 (39.5)	10 (38.5)	0.99
Sore throat	35 (31.3)	26 (30.2)	9 (34.6)	0.86
Chills	33 (29.5)	27 (31.4)	6 (23.1)	0.57
The average number of saliva samples per patient	3.6	3.7	3.4	0.13
Time from onset of symptoms to inclusion, in days (median [Q1; Q3]) (m.d = 10)	2.0 [2.0; 4.0]	2.5 [2.0; 4.0]	2.0 [1.0; 4.0]	0.56
Time from onset of symptoms to last saliva sample, in days (median [Q1; Q3]) (m.d = 10)	8.0 [7.0; 10.0]	9.0 [7.0; 10.0]	7.5 [5.0; 9.0]	0.36
Cycle threshold value of the first saliva sample	24.0 [20.8; 27.3]	24.5 [21.3; 27.8]	22.5 [20.0; 26.0]	0.36

## Data Availability

A special request can be addressed to rs-data@sentiweb.fr.
